# *Lupinus mutabilis* Edible Beans Protect against Bacterial Infection in Uroepithelial Cells

**DOI:** 10.1155/2018/1098015

**Published:** 2018-12-16

**Authors:** Witchuda Kamolvit, Vera Nilsén, Silvia Zambrana, Soumitra Mohanty, Eduardo Gonzales, Claes-Göran Östenson, Annelie Brauner

**Affiliations:** ^1^Department of Microbiology, Tumor and Cell Biology, Division of Clinical Microbiology, Karolinska Institutet and Karolinska University Hospital, 17176 Stockholm, Sweden; ^2^Department of Molecular Medicine and Surgery, Karolinska Institutet and Karolinska University Hospital, 17176 Stockholm, Sweden; ^3^Area de Farmacologia, Instituto de Investigaciones Farmaco Bioquimicas, Facultad de Ciencias Farmacéuticas y Bioquimicas, Universidad Mayor de San Andres, La Paz, Bolivia

## Abstract

*Lupinus mutabilis* is a South American herb with edible beans, known to reduce serum glucose levels in diabetic patients. Furthermore,* L. mutabilis* contains phytochemicals known to decrease bacterial load. Based on the increased urinary tract infections experienced among patients with diabetes, we investigated the effect of* L. mutabilis *on bladder epithelial cells in the protection of* E. coli *infection during normal and high glucose concentrations. We did not observe any direct antibacterial effect by* L. mutabilis* extract. Instead we observed an influence on the host cells, with indirect impact on bacteria and their possibility of causing infection.* L. mutabilis *extract decreased adhesion to bladder epithelial cells of uropathogenic bacteria, including drug-resistant strains. Moreover, uroplakin1a, involved in adhesion, was downregulated while the antimicrobial peptide RNase 7 was upregulated in* L. mutabilis* treated cells irrespectively of glucose concentration. This supports an early effect fighting bacteria. Additionally,* L. mutabilis* prevented bacterial biofilm formation, which is used by bacteria to evade the immune system and antibiotics. In summary,* L. mutabilis *protects against bacterial infection in uroepithelial cells by preventing adhesion through alteration of the cell surface, increasing antimicrobial peptide expression, and reducing biofilm formation. Together, this promotes bacterial clearance, suggesting that* L. mutabilis *as extract or as a dietary item can contribute to the prevention of urinary tract infections, which is of importance in an era of increasing antibiotic resistance.

## 1. Introduction

Urinary tract infection (UTI) is one of the most common infections, with* E. coli* as leading etiological agent [1]. The frequency of recurrent infections is high and consequences, aside from vast medical expenses, include considerable suffering—especially among patients with chronic diseases. In fact, patients with diabetes run a tenfold higher risk of contracting UTI compared to healthy volunteers [2]. The reason is not yet fully understood but can partly be explained by glycosuria promoting bacterial growth, autonomic neuropathy in the bladder and urethra, as well as antimicrobial peptide expression deficiency [3–5]. A therapy that can target this vulnerable and increasing group of patients is important.

When bacteria infect the urinary bladder, they adhere to the uroepithelial cells by attaching to adhesion factors of the host cells [6]. Simultaneously the innate immune response is activated with production of antimicrobial peptides, cytokines, and chemokines. Antimicrobial peptides belong to a group of diverse, positively charged peptides that disrupt bacterial membranes by binding to them. Contrary to antibiotics where resistance frequently develops, resistance to antimicrobial peptides is rare [7]. To avoid the immune system, bacteria hide within bacterial biofilm or intracellularly, where the accessibility for antimicrobial therapy is decreased.

The emerging global antibiotic resistance calls for immediate action, and therefore traditional herbal therapies constitute an area of increasing research interest. To prevent infection, studies have focused on antivirulence factors of uropathogenic* E. coli, *by enhancing the endogenous immune response with substances such as traditional herbs and vitamins [8–10].

*Lupinus mutabilis* is part of the Lupinus family with over 200 different species, originally from the Andes, Bolivia. It is famous for its edible beans, traditionally used to secure dietary protein intake. The beans contain a high number of phytochemicals, triglycerides, proteins, and alkaloids, e.g., lupanine [11]. Lupanine has been shown to stimulate insulin secretion in a glucose-dependent manner in mice, and blood glucose lowering properties have been observed in dysglycemic persons [12, 13]. Immune modulating properties have not been explored for* L. mutabili*s but their complex phytochemical components warrant further investigation and a potential role in preventing infections, e.g., UTI. Based on the antidiabetic effect of* L. mutabilis*, we investigated its effect on bladder infection in high glucose conditions, to mirror the diabetic condition.

The aim of this study was to examine the potential effects of* Lupinus mutabilis* extract on the interaction of uropathogenic bacteria and bladder epithelial cells using an* in vitro* model. We first investigated the bactericidal and biofilm preventing effects. Next the effect on the host antimicrobial peptide both under normo- and hyperglycemic conditions was studied.

## 2. Materials and Methods

### 2.1. Plant Material and Extraction

Plant specimen was collected from local producers from Ancoraimes Municipality, Omasuyos Province, La Paz, Bolivia (latitude 15°55′19.3′′S and longitude 68°53′50.1′′W). One voucher specimen (No. EG-1, Fabaceae) was identified and certified by the Herbario Nacional de Bolivia from Universidad Mayor de San Andrés (UMSA) and has been deposited in the Department of Pharmacology at the Instituto de Investigaciones Farmaco Bioquimicas, UMSA, La Paz, Bolivia.* L. mutabilis* seeds (200 g) from the plant specimen were powdered and macerated in 70% ethanol solution for 48 h to prepare the hydroethanolic extract (250 ml). To maximize the yield, the maceration procedure was repeated 5 times. Ethanol solvent was evaporated using a rotary evaporator (Heidolph, Schwabach, Germany) and the water fraction was dried under pressure in a freeze dryer (Labconco, Kansas City, MO, USA). Crude extracts obtained had an appearance of a yellow light powder with a yield of 22.0% w/w. For experiments the extract was dissolved in distilled water, and stock solutions were sterilized by a 0.22 *μ*m Millipore filter prior to use.

### 2.2. Bacterial Strains

*Escherichia coli* No. 12, isolated from a child with acute pyelonephritis, was used for all infection experiments. It expresses type 1 fimbriae and is able to form biofilm [14]. Additionally, the following bacteria were used:* Escherichia coli* (ATCC 25922), ESBL (Extended Spectrum Beta-Lactamase) producing* Escherichia coli* (CCUG 55971)* Klebsiella pneumoniae* (ATCC 13883), multidrug-resistant (MDR)* Klebsiella pneumoniae *(CCUG 58547),* Proteus mirabilis* (ATCC 29245),* Pseudomonas aeruginosa* (ATCC 27853),* Enterococcus faecalis* (ATCC 29212),* Staphylococcus saprophyticus *(ATCC 15305), and* Streptococcus agalactiae* (ATCC 13813). Prior to use, the bacteria were cultured at 37°C on Luria-Bertani (LB) or blood agar plates overnight.

### 2.3. Epithelial Cell Cultures

Human bladder epithelial cell lines T24 (HTB-4, ATCC) and 5637 (HTB-9, ATCC) were maintained in McCoy's 5A medium or RPMI 1640 (Gibco, Life Technologies, Paisley, UK), respectively, and supplemented with 10% fetal bovine serum (FBS) at 37°C with 5% CO_2_. T24 and 5637 were also maintained in RPMI 1640 medium, no glucose (Gibco, Life Technologies, Paisley, UK), and supplemented with 10% FBS and 5 mM glucose (Sigma-Aldrich, Schnelldorf, Germany). After overnight seeding into 24- or 96-well plates, cells were treated with 1000 *μ*g /ml of* L. mutabilis* extract and supplemented with desired concentration of glucose (5 mM for normoglycemia and ≥11 mM for hyperglycemia) 24 h prior to infection assay. Cells grown with medium alone served as controls.

### 2.4. Cell Viability Assay

The effect of* L. mutabilis* treatment on the viability of T24 and 5637 cells was determined at various concentrations and for different time points to evaluate the long term effect, in both cases using the XTT assay. Cells were treated with* L. mutabilis* extract at concentrations ranging from 300 to 20000 *μ*g/ml for 24 h and for 24 h, 48 h, and 72 h with 1000 *μ*g/ml* L. mutabilis *extract and then incubated with 250 *μ*l of 1 mg/ml XTT (Sigma-Aldrich, Schnelldorf, Germany) and 12.5 *μ*M menadione (Sigma-Aldrich, Schnelldorf, Germany) for 2 h according to the manufacturer's instructions. The conversion of tetrazolium salt XTT to a colored formazan derivative was measured at 490 nm in a 96-well plate. Nontreated control cells were maintained throughout the cell viability assay.

### 2.5. Adhesion and Invasion Assay

To evaluate the impact of* L. mutabilis *on the ability of uropathogens to adhere to uroepithelial cells, adhesion and rate of invasion assays were performed by infecting cells with 10^6^ of the previously mentioned bacteria, respectively, for 30 min in 37°C at 5% CO_2_. Thereafter, cells were washed with PBS to remove nonadherent bacteria. To collect cell associated bacteria, cells were lysed with 0.1% triton-X-100 in PBS. Lysates were plated on blood agar, and bacterial numbers were calculated after overnight incubation of serial dilutions. In the invasion assay, cells were infected and treated as in the adhesion assay. Thereafter fresh medium was added for 1 h, followed by exposure to gentamicin 100 *μ*g/ml for 30 min to kill extracellular bacteria. Finally, cells were washed with PBS, lysed, and plated as described for adhered bacteria. In treated cells, medium was supplemented with* L. mutabilis *throughout the entire experiment. Invasion rate was calculated by number of intracellular bacteria in relation to the total number of the adhered bacteria from the same experiment.

### 2.6. Antimicrobial Activity Assay

Direct antimicrobial activity of* L. mutabilis *was determined against the following bacteria:* E. faecalis* (ATCC 29212),* E. coli* (ATCC 25922),* K. pneumoniae* (ATCC 13883),* P. mirabilis* (ATCC 29245),* P. aeruginosa* (ATCC 27853),* S. saprophyticus *(ATCC 15305), and* S. agalactiae* (ATCC 13813). The minimum inhibitory concentration (MIC) of* L. mutabilis *was determined using a broth microdilution method in 96-well polystyrene microtiter plates according to the Clinical Laboratory Standards Institute (2016) guidelines, with some modifications. The stock solution of extract was serially twofold diluted in cationic adjusted Mueller Hinton broth (MHB) and the final concentration ranged from 50 to 0.2 mg/ml. The final inoculum was 5 × 10^5^ CFU/ml.

### 2.7. Crystal Violet Assay

To investigate* L. mutabilis* extract effect on bacterial biofilm formation, crystal violet assay was used.* E. coli* No. 12 (5 × 10^4^ CFU/ml) was grown in LB broth without salt, supplemented with 0, 5, and 11 mM glucose concentrations, and treated with* L. mutabilis* at concentrations of 400 and 1000 *μ*g/ml. After incubation at 37°C with 5% CO_2_ for 72 h, planktonic cells were removed, and wells were washed with PBS twice and then stained for 10 minutes with 0.3% crystal violet. After removing crystal violet and washing with tap water, a 1:5 solution ethanol and acetone was used to dissolve the remaining crystal violet stains from the biofilm formation. After shaking wells for 10 min in 200 rpm, the OD was analyzed using spectrophotometry at 570 nm.

### 2.8. Total RNA Extraction, cDNA Synthesis, and Gene Expression Analysis

Total RNA was extracted from bladder epithelial cells using the RNeasy Mini Kit (Qiagen, Hilden, Germany) according to the manufacturer's protocol. Concentration and purity of RNA were determined using Nanodrop, and up to 1 *μ*g of RNA was transcribed to cDNA using the High-Capacity cDNA Reverse Transcription Kit (Applied Biosystems, Vilnius, Lithuania) [15]. Real-time PCR for caveolin-1 (*CAV1*, Hs00184697_m1), *β*1 integrin (*ITGB1*, Hs00559595_m1), and uroplakin1a (*UPK1A*, Hs00199638_m1) was analyzed using specific TaqMan gene expression assays (Applied Biosystems, Vilnius, Lithuania) in a Rotor-Gene PCR cycler (Corbett Life Science, Hilden, Germany). Human GAPD (*GAPDH*, 4326317E) and 18S (*18s*, Hs03003631_g1) were used as housekeeping controls. Furthermore, the antimicrobial peptides human cathelicidin LL-37 (*CAMP*, Hs00189038_m1) and psoriasin (*S100A7*, Hs00161488_m1); human *β*-defensin 2,* DEFB4A*_F (5′- ccctttctgaatccgc) and* DEFB4A*_R (5′- gagggtttgtatctcct); RNase 7,* RNASE7*_F (5′- ggagtcacagcacgaagacca) and* RNASE7*_R (5′- catggctgagttgcatgcttga) were analyzed using SYBR® Green specific primers. *β*-actin,* ACTB*_F (5′-aagagaggcatcctcaccct), and* ACTB*_R (5′-tacatcgctggggtgttg) served as housekeeping controls for comparison of cycle values. Relative expressions of target genes were presented as 2^-ΔCT^ and fold change as 2^-ΔΔCT^ compared to uninfected and nontreated control.

### 2.9. Immunofluorescence Staining of Cells

5637 cells were treated with 1000 *μ*g/ml of* L. mutabilis* for 24 h in both 5 mM and 11 mM cells. Cells were fixed in 4% PFA for 30 min at room temperature and permeabilized with 0.1% triton-X-100 in PBS. Permeabilized cells were blocked with 5% BSA in PBS for 30 min at room temperature. Then cells were incubated overnight at 4°C with RNase 7 primary antibody (1:200, Icosagen) or uroplakin1a (1:200, Santa Cruz) in 1:1 ratio of 1 X PBS with 0.1% Tween 20 (PBS-T) and 5% BSA in PBS. Cells were washed with 1 X PBS-T and further incubated with secondary Alexa Fluor–conjugated antibody (1:500, Invitrogen) for 1 h at room temperature and mounted in Fluoromount G (Southern Biotech). Slides were analyzed with a Leica SP5 confocal microscope using 63X oil immersion objective, from random view fields per coverslip.

### 2.10. Statistical Methods

Statistical analyses were performed using Graph Pad Prism Version 5.04 (GraphPad Software, San Diego, CA, USA). For comparison between treated and control groups, Student's unpaired t-test, one-way ANOVA, and post hoc Bonferroni test were used. Differences were considered significant if p-value = 0.05 or less.

## 3. Results

### 3.1. Effect of* L. mutabilis* on Uroepithelial Cells and Direct Antibacterial Properties on Common Uropathogenic Bacteria

Cell viability assay using XTT confirmed that* L. mutabilis* extract at concentrations up to 1000 *μ*g/ml did not affect the viability of bladder epithelial cells, which remained 99% after 24 h of treatment. When increasing the concentration to 20 mg/ml, the cell survival decreased to approximately 80% (Supplementary Figure 1(A)). A timescale cell growth assay was also performed up to 72 h with and without the extract, not showing any difference in growth pattern of* L. mutabilis* treated cells compared to untreated cells (Supplementary Figure 1(B)). This confirmed that* L. mutabilis* did not arrest the cell cycle nor affect vital aspects of cell metabolism. Furthermore,* L. mutabilis *extract did not have any bactericidal effect, when tested up to 125 mg/ml on* E. coli, K. pneumoniae, P. mirabilis, P. aeruginosa*,* S. saprophyticus*, and* E. faecalis*.

### 3.2. *L. mutabilis* Extract Decreases Adhesion of Uropathogenic Bacteria

**Figure 1:**
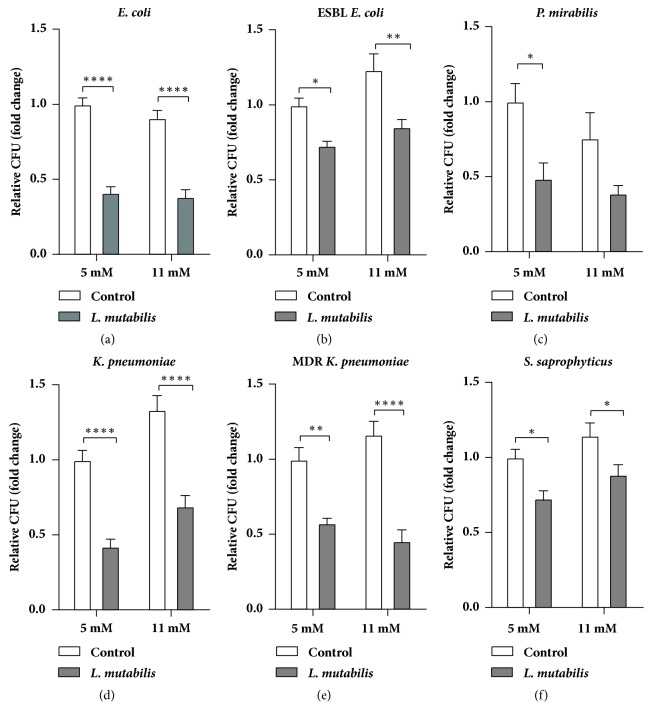
Adhesion assay. Pretreatment with* L. mutabilis* inhibits bacterial adhesion in bladder epithelial cells in 5 mM and 11 mM glucose concentrations. Adhesion assay was performed on T24 cells after prior treatment with 1000 *μ*g/ml of* L. mutabilis* for 24 h, followed by 30 min infection with the following bacteria:* E. coli* (a), extended spectrum beta-lactamase (ESBL)* E. coli* (b),* P. mirabilis* (c),* K. pneumoniae* (d), multidrug-resistant (MDR)* K. pneumoniae* (e), and* S. saprophyticus* (f). Data shown are +/- SEM. Results were normalized to nontreated uninfected control cells indicated by P*∗* <0.05, P*∗∗* <0.01, and P*∗∗∗∗* <0.0001. Data were calculated from at least two independent experiments with triplicates per condition. MDR refers to strain that is resistant to three or more antimicrobial classes.

After excluding that* L. mutabilis *had direct, bactericidal effect on uropathogenic bacteria, we investigated the possible indirect effector mechanisms. Adhesion is the critical, first step of infection in uroepithelial cells [16]. We observed that 1000 *μ*g/ml treatment of* L. mutabilis *extract for 24 h prior to the infection significantly decreased the ratio of bacteria adhering to T24 bladder epithelial cells both at 5 and 11 mM glucose, reflecting normoglycemic and hyperglycemic conditions. Such decrease was observed when testing the common uropathogenic bacteria* E. coli*, ESBL producing* E. coli, K. pneumoniae*, multidrug-resistant* K. pneumoniae*,* P. aeruginosa*, and* S. saprophyticus* (Figure 1). Moreover,* L. mutabilis* treatment significantly decreased the adhesion of* P. mirabilis* in normoglycemia. The invasion rate of the same uropathogens was not significantly changed after* L. mutabilis* treatment (data not shown).

### 3.3. *L. mutabilis* Alters Cell Surface Proteins and Expression of Antimicrobial Peptides in Uroepithelial Cells

**Figure 2:**
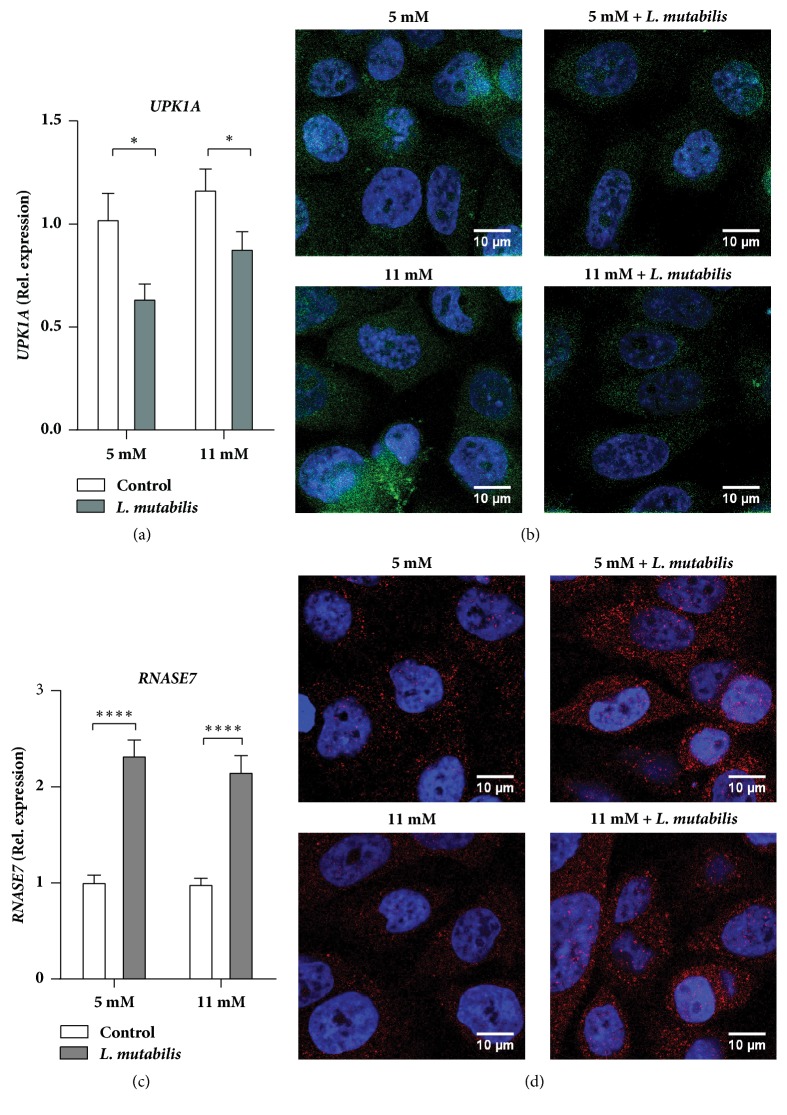
mRNA and protein expression of cell surface receptor and antimicrobial peptides in uninfected uroepithelial cells. Expression of* UPK1A* (a) and* RNASE7* (c) at mRNA level in cells treated for 24 h with 1000 *μ*g/ml of* L. mutabilis* in 5 and 11 mM glucose and nontreated cells in equivalent glucose concentrations as control. Immunofluorescence staining also confirmed downregulation of UP1a (b) and upregulation of RNase 7 (d) after 24 h of 1000 *μ*g/ml of* L. mutabilis* treatment in both 5 and 11 mM cells when compared to respective control cells. Data shown are +/- SEM. Results were normalized to nontreated uninfected control cells indicated by P*∗* <0.05 and P*∗∗∗∗* <0.0001. Expression of mRNA was from a total of three independent assays with duplicates per condition.

One of the most common indirect antibacterial mechanisms, observed with the use of herbal extracts, such as* C. bolivianum*, is downregulation of cell surface proteins to which bacteria bind when adhering to the host cells [10]. The effects of uroplakin1a (*UPK1A*), *β*1 integrin (*ITGB1*), and caveolin-1(*CAV1*) were therefore investigated. We observed that the* UPK1A* mRNA was significantly decreased in pretreated uroepithelial cells (Figure 2(a)) with a similar trend on the protein level (Figure 2(b)), both at 5 and 11 mM glucose. However,* L. mutabilis *pretreatment did not influence caveolin-1 or *β*1 integrin expression (data not shown).

Interestingly, the antimicrobial peptide,* RNASE7 *mRNA, was significantly increased in bladder cells pretreated with* L. mutabilis* both in normal and high glucose concentrations (Figure 2(c)), with similar results on the protein level (Figure 2(d)). Expression of the antimicrobial peptides cathelicidin, LL-37 (*CAMP*), and human *β*-defensin 2, hBD-2 (*DEFB4A*), at mRNA remained unchanged in the pretreated cells (data not shown).

### 3.4. *L. mutabilis *Extract Inhibits* E. coli* Biofilm Formation

**Figure 3:**
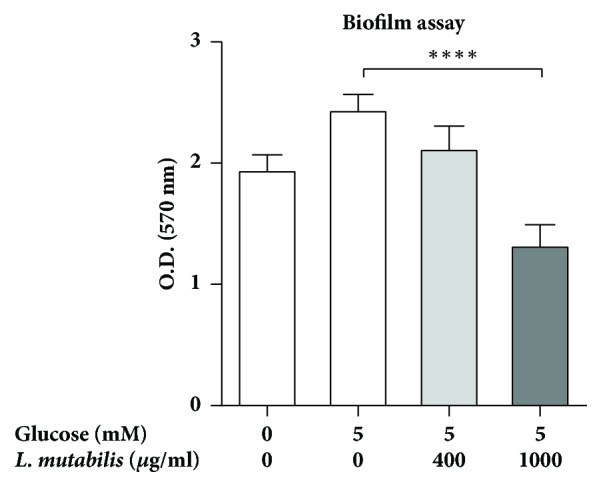
*E. coli* No. 12 biofilm adherence and thickness. Bacteria and* L. mutabilis* extract were added to LB medium without salt with supplementation of 0 mM and 5 mM of glucose, respectively. Bacteria were incubated with 5% CO_2_ for 72 h before being washed and stained with crystal violet for spectrophotometry. Data shown are mean +/- SEM. *∗∗∗∗*P < 0.0001. Biofilm assays were calculated from at least two independent experiments with six replicates per condition.

*E. coli* forms biofilm as protection against innate immune responses and antibacterial therapies. Thereby bacteria become less accessible for antibiotics and may persist either extracellularly or in the uroepithelial cells as intracellular bacterial communities, possibly reoccurring at a later time point [16]. Although* L. mutabilis *did not have bactericidal effect, we observed that treatment with* L. mutabilis* extract at concentration, 1000 *μ*g/mL, significantly reduced biofilm formation at normal glucose conditions, 5 mM (Figure 3). However, in higher glucose concentrations, 11 and 30 mM, no significant effect of* L. mutabilis *was observed.

## 4. Discussion

Herbal extracts are frequently used in traditional medicine, although the underlying mechanisms are not always known. We here demonstrate that extract from* L. mutabilis* does not exhibit bactericidal effect. However, by blocking bacterial adhesion to the host cell,* L. mutabilis* efficiently prevents infection [16]. The decreased adhesion is due to downregulation of the cell surface protein, uroplakin1a, which is pivotal for the initiation of bacterial infections in the urinary tract; this is similar to our earlier finding where two other plant extracts,* Clinopodium bolivianum* and* Amaranthus caudatus*, also decreased the adhesion and invasion by downregulating uroplakin1a [10, 17]. On the contrary, no effect was observed on caveolin 1 or *β*1 integrin, both of them associated with invasion to host cells [16], supporting the assumption that* L. mutabilis* pretreatment solely acts on bacterial adherence. Bacteria are equipped with adhesins, out of which the type 1 fimbrial adhesin, FimH, is necessary for adhesion to occur. It binds to uroplakin1a, which therefore is crucial for the infection process [18]. Because of its importance, FimH has attracted much attention. Blocking the effect of the FimH antigen using vaccine [19], or a molecule that specifically blocks FimH binding to the host, e.g., mannose has successfully been tried in mice and humans [20, 21]. Here we demonstrate a possible alternative mode of action, instead targeting the cell surface protein, uroplakin1a.

Antimicrobial peptides kill bacteria extracellularly before they adhere to the epithelium [22]. Different plant extracts are known to upregulate host antimicrobial peptides; e.g.,* A. paniculata* upregulates human *β*-defensin 2 in lung epithelial cells [23]. Interestingly, the antimicrobial peptide RNase 7 was upregulated in* L. mutabilis* pretreated cells, contrary to LL-37 and human *β*-defensin 2. This indicates that pretreatment with* L. mutabilis *specifically targets RNase 7 to fend off bacteria adhering to the uroepithelial cell surface. Interestingly, RNase 7 is naturally expressed in uroepithelial cells and is known to have bactericidal effect on Gram-negative and Gram-positive bacteria as well as MDR strains [24–26], which is in line with our results. Decreased bacterial adhesion with* L. mutabilis* pretreatment as well as upregulation of RNase 7 expression was seen both in normal and hyperglycemic conditions, mimicking metabolic conditions in the urinary bladder of diabetic patients. This finding is of clinical interest because diabetic patients are known to have an impaired function of antimicrobial peptides such as RNase 7 due to lack of insulin [5].

Moreover, we observed that* L. mutabilis* reduces* E. coli* biofilm formation without inhibiting bacterial growth, suggesting an independent effect on biofilm formation. These findings are in line with our previous findings that subinhibitory concentrations of LL-37 reduced* E. coli *biofilm formation [14].

Chemical analysis of the* L. mutabilis *extract used in our study shows high triglyceride content and confirmed the presence of several bis-quinolizidine alkaloids, e.g., sparteine, lupanine, oxylupanine, 11,12-dehydrolupanine, and nuttalline [27], which is partly in agreement with previous data [11].

A limitation of the study is that the extract of* L. mutabilis *was used and not the different identified substances. Due to the complexity of the chromatogram, we decided to show a possible effect of the extract. It is likely that a combination of different already characterized and not yet characterized substances will be needed to reach optimal effect.

## 5. Conclusions

In conclusion, our* in vitro* studies suggest that* L. mutabilis* prevents infections of uropathogenic bacterial strains, irrespective of glucose concentrations. This effect is mediated by downregulation of the cell surface protein, uroplakin1a, and upregulation of the antimicrobial peptide RNase 7. Furthermore, bacterial biofilm formation is decreased, which may enhance the antibiotic effect. Taken together, pretreatment with extracts from* L. mutabilis* or* L. mutabilis *beans as a functional food item may in the future be used to prevent UTI in patients at increased risk. Future work will aim to study* in vivo* effect of* L. mutabilis *by using murine urinary tract infection model.

## Data Availability

All the data supporting the results reported in the published article will be made available.

## Supplementary Materials

Supplementary Materials Figure 1. Cytotoxic activity of* L. mutabilis* on bladder epithelial cells. (A) XTT assay was performed in T24 cells after treatment with* L. mutabilis* for 24 h. Viability of treated cells were compared with respect to untreated control cells. (B) Timescale growth curve analyses of 5637 cells were performed up to 72 h and the viability was compared to the untreated control cells. Data shown are mean ± SEM, from four independent experiments, performed in triplicate. *∗∗∗∗* P < 0.0001.
