# Supplementary material for: Lupinus mutabilis Edible Beans Protect against Bacterial Infection in Uroepithelial Cells
Source: Evid Based Complement Alternat Med. 2018 Dec 16;2018:1098015. doi: 10.1155/2018/1098015 (PMC6311276; doi:10.1155/2018/1098015)
Supplement: Supplementary Materials — Figure 1. Cytotoxic activity of L. mutabilis on bladder epithelial cells. (A) XTT assay was performed in T24 cells after treatment with L. mutabilis for 24 h. Viability of treated cells were compared with respect to untreated control cells. (B) Timescale growth curve analyses of 5637 cells were performed up to 72 h and the viability was compared to the untreated control cells. Data shown are mean ± SEM, from four independent experiments, performed in triplicate. ∗∗∗∗ P < 0.0001. [file 1098015.f1.zip › supplementary_fig_1_ecam_2600071.docx]

Supplementary Fig 1.

Fig 1. Cytotoxic activity of L. mutabilis on bladder epithelial cells. (A) XTT assay was performed in T24 cells after treatment with L. mutabilis for 24 h. Viability of treated cells were compared with respect to untreated control cells.

(B) Timescale growth curve analyses of 5637 cells were performed up to 72 h and the viability was compared to the untreated control cells. Data shown are mean ± SEM, from four independent experiments, performed in triplicates. **** P < 0.0001.
